# [Corrigendum] Interpretation of HbA1c lies at the intersection of analytical methodology, clinical biochemistry and hematology (Review)

**DOI:** 10.3892/etm.2023.11885

**Published:** 2023-03-13

**Authors:** Zixin Chen, Limei Shao, Mingfeng Jiang, Xuejiao Ba, Bingjie Ma, Tao Zhou

Exp Ther Med 24:707, 2022; DOI: 10.3892/etm.2022.11643

Subsequently to the publication of the above article, an interested reader drew to the authors’ attention that there were a number of potential errors distributed throughout the text that were likely to have distorted the sense of what the authors were intending to convey. After having consulted the authors about these points, it was confirmed that there were certain aspects of the review that were in need of correction / clarification.

This corrigendum therefore aims to rectify the problem of the incorrect factual information presented in this review, as follows (changes to the published text are highlighted in bold):

i) In the section “**3. Biochemistry of hemoglobin A1c**” on p. 2, line 7, the sentence starting here should have read as: “In the presence of glucose, **glycan** interacts with the amino group of Hb.”;

ii) In the same section, on line 10, the sentence starting here should have read as: “The first step is the reaction of the aldehyde group of glucose with the NH_2_ group of the amino acid to form a Schiff base or **aldimine**,...”;

iii) In the same section, the sentence concluding on line 16 should have read as, “... which contains a more stable and irreversible ketoamine bond, **yielding** HbA1c.”

iv) In the section “**4. HbA1c detection methods**”, towards the bottom of the left-hand column of p. 2, the first sentence in the second paragraph should have simply read: “The boronate affinity chromatography (BAC) method is often used as a reference method in several studies.” (it was incorrect to have stated that “it can reveal the presence of Hb variants with minimal analytical interference.”);

v) finally, where the term “glycosylation” was featured in this review (especially in the subsection “*Drugs*” in the right-hand column of p. 6), the authors wish to clarify that they were referring throughout to “**non-enzymatic** glycosylation (otherwise, known as “glycation”), as was specifically stated in line 8 of this subsection.

The authors thank the interested reader for bringing this matter to their attention. All the authors agree with the publication of this corrigendum, and are grateful to the Editor of *Experimental and Therapeutic Medicine* for granting them the opportunity to publish this; furthermore, they apologize to the readership for any misunderstandings or confusion caused.

